# Sports wagering in the context of addictive disorders: results from a census-matched U.S. sample

**DOI:** 10.1080/28324765.2023.2231497

**Published:** 2023-07-11

**Authors:** Joshua B. Grubbs, Shane W. Kraus

**Affiliations:** a Center for Alcohol, Substance use, And Addictions and Department of Psychology, University of New Mexico, Albuquerque, USA; b Department of Psychology, University of Nevada, Las Vegas, USA

**Keywords:** sports gambling, fantasy sports, daily fantasy, gaming, substance use, pornography

## Abstract

Recent legal cases, technological advances, and sports league administrative decisions have provided unprecedented access to various forms of sports wagering across the United States (U.S.). Such increases in access have highlighted gaps in current understanding of how sports wagering is related to a range of addictive behaviors. The present work explores how diverse forms of sports wagering are related to engagement in and symptoms of addiction to a variety of behaviors and substances. Participants were recruited by YouGov Opinion polling and taken from two U.S. samples. Sports-wagering habits, substance use patterns, and self-reported symptoms of addiction to behaviors and substances were assessed. Across behaviors measured, sports-wagering was consistently associated with greater engagement in the use of diverse substances and potentially addictive behaviors such as gaming and pornography use. Self-reported symptoms of addiction to such substances and behaviors were consistently and uniquely related to greater reports of sports wagering, except for traditional fantasy sport play. Collectively, these results suggest that sports wagering behaviors, especially traditional sports wagering, e-sports wagering, and taking part in daily fantasy leagues are often linked to a wide variety of impulsive or at-risk behaviors and may be seen as an indicator of more broad concerns with risky behaviors.

1.

Gambling has existed as a popular recreational activity for millennia (Schwartz, 2013), with records of gambling on sporting events existing for comparable amounts of time (Laurence et al., 2011). Even so, sports wagering has evolved over recent decades, due to increased legalization of sports wagering, the proliferation of modern sports leagues, and technological advances in sports broadcasting, sports analysis, and sports betting. In the United States (U.S.), since 2018, almost three dozen states have either legalized or passed legislation that will ultimately legalize a variety of sports wagering activities (American Gaming Association, 2022). Whereas sports wagering was previously confined to very specific geographical locations in the U.S., the Supreme Court of the United States’ ruling in Murphy v. National Collegiate Athletic Association (*Murphy v. National Collegiate Athletic Association*, 2018) spurred increased access to this type of gambling. Combined with technological advances in the gambling industry over recent decades, it is not an overstatement to conclude that the average U.S. resident now has unprecedented access to this activity.

Despite such rapid increases in access to sports betting, research in the U.S. related to sports betting has lagged, prompting a great deal of public facing concern about the public health implications of this type of gambling and the potential risks it might pose (J. B. Grubbs & Kraus, 2022; Nower, 2023). Such concerns highlight a clear need for more careful research related to sports wagering and other health behaviors, such as addictive disorders. To address this need, the present work seeks to understand the relationships between sports gambling behaviors and a range of potentially addictive behavior patterns, with a goal of highlighting relationships between these domains and providing general insights into how such relationships might impact clinical care and community prevention efforts.

## 2. Sports betting in the 21^st^ century

As mentioned above, the 21^st^ century has brought greater legal access to sports betting in the U.S. However, these changes have not simply been in legal access. Technological advances have dramatically shifted how people approach sports gambling activities. Mobile betting applications continue to rise in popularity. As of July 2022, with mobile sports wagering legal in 20 states, millions of Americans can now legally engage in sports wagering from the comfort and privacy of their own homes via a range of internet-connected devices (American Gaming Association, 2022).

The past several years have also witnessed the rise of fantasy sports leagues across almost all major U.S. sports (Pickering et al., 2016). These leagues allow participants to compile hypothetical teams of athletes within a given sport, with athletes’ individual performances contributing to the hypothetical team’s score in each week of the fantasy sport season. These hypothetical teams then, most often, are pitted against another participant’s hypothetical team, with the team achieving the highest weekly score winning the matchup. Though free fantasy sports leagues exist, most fantasy sports require buy-in or access fees, with participants competing over the course of the fantasy season in the hopes of winning money or prizes. In short, participants wager money in the hopes of winning more money based on the outcome of the fantasy play. By U.S. federal law, fantasy sports are considered games of skill, rather than chance. Therefore, fantasy sports are legal throughout the U.S. and are explicitly endorsed by many major sporting leagues. Even so, the similarities between fantasy sport play and sports wagering are well-documented (Nower et al., 2018). Moreover, *daily* fantasy sport leagues have risen sharply in popularity, as these leagues allow players to draft new teams each week and compete for cash prizes at an accelerated rate, potentially winning (or losing) money several times in a short time span (Billings et al., 2017).

Moving beyond traditional sports wagering behaviors and fantasy sports, competitive video-game play, termed electronic sports (e-sports), has increased in recent years (Gainsbury et al., 2017; Greer et al., 2021). In the U.S., at both professional and collegiate levels, e-sports continue to grow, with increasing interest and investment in these sports from younger generations. Not surprisingly, as the popularity of these new competitive endeavors has risen in the past decade, gambling on e-sports has also risen, presenting a new form of sports wagering with a small but increasing market share that has been largely unexplored in prior gambling research. What little research that does exist in this domain suggests that wagering on e-sports may be particularly associated with greater symptoms of gambling disorder (Greer et al., 2021; Peter et al., 2019). Indeed, recent work on national U.S. samples has shown e-sports wagering to be the form of sports wagering that is most powerfully predictive of symptoms of gambling disorder, beyond the effects of traditional sports wagering or fantasy sports play (J. B. Grubbs & S. W. Kraus, 2022).

Internationally, there is a well-established body of research related to sports wagering (Etuk et al., 2022). Research from the United Kingdom has found that sports betting is longitudinally associated with greater problem gambling symptoms and greater financial expenditures among gamblers (Wardle et al., 2022). Similarly, a number of studies from Australia have highlighted that committed sports bettors appear to engage in sophisticated betting strategies (Gainsbury & Russell, 2015) and that high-risk sports bettors (i.e., those that are particular risk of gambling related problems) are often more impulsive, more deeply engaged with sports betting, and more likely to endorse financial motives for their gambling behaviors (Hing et al., 2016; Russell, Hing, & Browne, 2019; Russell, Hing, Li, et al., 2019). Provincial studies in Ontario, Canada have shown that sports bettors, when compared to non-sports betting gamblers, are more likely to be younger, to be male, to identify as a gambler, and to demonstrate inaccurate beliefs about gambling (Cooper et al., 2021). Even so, there have been few recent studies have been conducted at the population level in the U.S (Marchica et al., 2017) and understanding of links between sports wagering and problem gambling in the U.S. cultural context are sorely needed (Etuk et al., 2022).

## 3. Sports wagering and addictive behaviors

In considering how sports wagering relates to addictive disorders, a brief note about the current state of addictions research is warranted. Addictions and addictive behaviors have captured attention in psychiatric and mental health research and treatment efforts for decades. Over that time, numerous debates about the true nature of addiction and what behaviors or activities might be considered as addictions have ebbed and flowed (West & Brown, 2013). Yet, controversies about the nature of addiction have become more intense in recent years owing, in large part, to the expansion of addictive disorders to include non-substance addictions in both the *Diagnostic and Statistical Manual of Mental Disorder* (DSM-5) and the eleventh edition of the *International Classification of Diseases* (ICD-11). In both manuals, gambling disorder is now recognized as an addictive disorder (Fauth-Bühler et al., 2017; Kim & Hodgins, 2019), and, in the ICD-11, gaming disorder (i.e., video game addiction) is also recognized as an addictive disorder (King & Potenza, 2019).

As alluded to above, these inclusions were controversial for a variety of reasons. A number of scholars have voiced concerns that current bodies of research related to behavioral addictions are not sufficiently developed to conclude whether such disorders are indeed addictions (Grubbs et al., 2020; Kraus et al., 2016; Petry et al., 2018). Other scholars have also suggested that expanding definitions of addiction to non-substance use disorders obscures distinctions between compulsive behaviors and true addictions (Aarseth et al., 2017). Similarly, there is also a seemingly reasonable fear that labeling non-substance behaviors as addictions presents potential to pathologize everyday life and largely normal behaviors (Billieux et al., 2015; Kardefelt‐Winther et al., 2017). Other studies have shown that common measures of so-called behavioral addictions are poorly constructed in ways that would lead almost any behavior to seem addictive, thereby impeding the ability to draw any reasonable inference about the behavior being assessed (Satchell et al., 2020). Additionally, some scholars have pointed out that the social and moral contexts of so-called addictive behaviors often obscure the ability to accurately study them in large-scale surveys (Grubbs, 2021). Even so, a growing body of research has consistently and vigorously contended that the recognition of behavioral addictions as true addictions is both empirically justified and diagnostically accurate (Clark, 2014; Clark & Limbrick-Oldfield, 2013; Grant et al., 2010).

In the context of the above controversies, the present work is not poised to settle any debates centered on deciding whether behavioral addictions are “true” addictions. Even so, for the present work, we will use the terms “addictive behaviors” and “potentially addictive behaviors” throughout to refer to both substance and non-substance related behaviors that might become excessive, compulsive, or out-of-control in ways that are consistent with an addiction. As is noted above, we acknowledge that there are deep controversies within the psychiatric, psychological, and mental health literatures as to the nature of what addictions are and whether non-substance behaviors can ever be considered as addictions. Yet, both gambling and video game play are now recognized by major diagnostic manuals as potential foci of addiction; accordingly, we have elected to use *addiction* related terminology to refer to such disorders. We also note that the use of this term is not meant as a statement about the etiology or mechanisms underlying these disorders, but rather as a *descriptive* term about the potential for some behaviors to become dysregulated, excessive, or compulsive in ways that are consistent with the general archetype of what is considered an addiction.

In considering possible links between sports wagering and addictive disorders, gambling disorder remains the most logical associate of such behaviors. Gambling disorder (GD) and problem gambling are long-recognized public health concerns (Korn & Shaffer, 1999), and excessive, compulsive, or addictive gambling behaviors have been classified as a distinct psychiatric illness (Fauth-Bühler et al., 2017) in both the American Psychiatric Association’s *DSM* (since 1980) and the World Health Organization’s *ICD* (since 1977). Though estimates of GD prevalence vary widely across studies (Loo et al., 2019) and range from under 1% to roughly 4% (Welte et al., 2015), scientists and clinicians generally agree that even conservative estimates of the prevalence of gambling disorder would indicate that there are hundreds of thousands of Americans who experience difficulties in regulating their gambling behaviors, leading to financial, relational, mental health, and legal consequences (Fauth-Bühler et al., 2017).

Not surprisingly, past research clearly demonstrates robust links between sports wagering and symptoms of GD (van der Maas et al., 2022; Winters & Derevensky, 2019). For example, sports wagering is linked to self-reported symptoms of GD, beyond the influence of other gambling behaviors such as electronic gaming machines and other forms of betting (LaPlante et al., 2014; Marchica et al., 2017). Similarly, fantasy sports play, daily fantasy sports play, and online sports wagering are all linked to greater report of problem gambling symptoms (Nower et al., 2018), lending support to the notion that specific forms of sports wagering may be of importance when assessing links between sports wagering and problematic gambling outcomes. Building on this, recent work in nationally representative U.S. samples has clearly shown that daily fantasy sports play, e-sports wagering, and traditional sports wagering are uniquely associated with problem gambling symptoms, though traditional fantasy sports play does not appear to be so related (J. B. Grubbs & S. W. Kraus, 2022).

Beyond GD alone, there is emerging evidence that sports wagering behaviors are likely to be associated with other addictive behaviors. Studies of U.S. adolescents (Marchica et al., 2017), Serbian adults (Todorovic et al., 2020), Australian adults (Russell, Hing, & Browne, 2019), and European adolescents (Molinaro et al., 2018) all show links between sports wagering generally and greater use of a variety of substances and greater symptoms of addiction to or dysregulation in substance use. In short, engagement with sports wagering appears to be related to engagement in a variety of other addictive behaviors, though only limited research has examined this in the U.S, which provided the impetus for the present study.

## 4. The present study

Despite compelling evidence to support the notion that sports wagering is an important form of gambling behavior, research in this domain (particularly in the U.S.) has not kept pace with ongoing American legalization efforts and wide expansion of sports betting. Though excellent studies on sports wagering behaviors have been conducted in the United Kingdom (Wardle et al., 2022), Australia (Gainsbury & Russell, 2015; Greer et al., 2021; Hing et al., 2016; Russell, Hing, & Browne, 2019), Canada (Cooper et al., 2021) and a range of other nations (Columb et al., 2020; Labrador & Vallejo-Achón, 2020), few recent studies have been conducted at the population level in the U.S (Etuk et al., 2022).

Moving further, the rapidly changing face of sports wagering behaviors in the U.S. and globally suggests that there is a great deal yet unknown about how sport wagering behaviors relate to other domains of interest, particularly with regard to substance use disorders and other potentially addictive behaviors. Combined with the growing body of evidence suggesting that different forms of sports wagering behaviors might be more or less problematic than other forms (i.e., general fantasy play vs. daily fantasy play), there is a clear need to better document how diverse forms of sports wagering are related to other domains of mental health outcomes (J. B. Grubbs & S. W. Kraus, 2022). Finally, given the previously established links of both gambling generally and sports wagering specifically with various addictive behaviors, there is strong reason to suppose that sports wagering might co-occur with other substance use disorders and addictive behavior problems.

In service of the above aims, we evaluated the links between sports wagering behaviors and a variety of addictive behaviors and symptoms of interest in a census-matched, U.S. national sample. In so doing, we sought to answer the following questions.

1. What are the associations between various forms of sports betting frequency (i.e., traditional fantasy sports play, daily fantasy sports play, traditional sports betting, and e-sports betting) and frequency of various other potentially addictive behaviors (i.e., substance use, pornography use, and gaming)?
2. What are the associations between various forms of sports betting frequency and self-reported feelings of addiction to or dysregulation in the above mentioned potentially addictive behaviors?

Of note, in each of the above cases, we sought to evaluate the links between these domains, but not to delineate causality. Accordingly, we focused on expected associations between behavioral domains, rather than directional claims. Broadly, we expected to find that sports wagering would be linked to greater self-reported frequency of other addictive behaviors, both substance and behavioral. Additionally, we hypothesized that the links between sports wagering and such addictive behaviors would vary based on the type of sports wagering being evaluated, with regular fantasy play being generally not associated with problematic outcomes, once daily fantasy play, traditional sports wagering, and e-sports wagering were all considered. Specifically, we expected to find that, of the four types of sports wagering discussed thus far (traditional sports wagering, fantasy sport play, daily fantasy sport play, and e-sports wagering), fantasy sport play would be the least associated with pathological outcomes. Given the novelty of this research, however, we did not speculate on the relative associations of the other three forms of sports wagering with other addictive behaviors.

## 5. Method

### 5.1. Participants and procedure

Participants were recruited via YouGov opinion polling. YouGov is an international polling, opinion, and marketing firm that supports active panels of over 17 million participants. Though YouGov is an opt-in service, their proprietary recruitment strategies and demographic weighting formulae consistently perform at rates comparable to or better than more traditional full-probability sampling vendors, resulting high-quality, representative samples (Kennedy et al., 2016; Rivers, 2016). Comparisons of YouGov with traditional non-probability and probability survey vendors (e.g., PEW research center), consistently find that the quality and representativeness of data from YouGov matches or exceed measures of data quality and indicators of representativeness among true probability vendors. Blinded analyses of non-probability and probability vendors (Kennedy et al., 2016), conducted by PEW Research, clearly showed that YouGov outperformed all other vendors, including PEW’s own probability sampling service. YouGov uses a sample-matching method to construct representative samples from a large online opt-in panel. For our work, the company drew on a random hypothetical sample based on the 2019 American community survey that corresponds to the sampling frame and selects panelists who match sampling frame members’ demographic characteristics. The matched cases were then weighted to the sampling frame using propensity scores. YouGov provided poststratification weights, based on age, race ethnicity, educational level, and both 2020 and 2016 vote history, when available. Advertisements for recruitment did not disclose the gambling emphasis of the study. That is, as some prior works have noted (Angus et al., 2021), demand characteristics in online surveys can lead to participants reporting behaviors they do not actually engage in so that they may participate in compensated surveys. Accordingly, this study was not framed as focusing on gambling behaviors in advertisements. Rather, the focus of survey advertisements was on participating in research related to recreational activities, personality, and mental health.

For the present work, we recruited two related samples. For the first sample, we recruited American adults (*N* = 2,806; *M*_*age*_*[SD]=*48.9[17.2]; 1365[48.6%] men; response rate = 87.6%) matched and weighted for U.S. representative norms for age, gender, education, census region, and race/ethnicity as of the 2019 American Community Survey (U.S. Census Bureau, 2019). For our second sample, we oversampled sports-wagering adults in the U.S. (*N* = 1,557; *M*_*age*_*[SD]=*41.7[15.3]; 1043[67%] men; response rate = 78.7%), matched to the demographics of sports wagering individuals from our first sample. For all analyses, we used a combined sample consisting of participants derived from both samples above. Full demographics for the total sample and by sports wagering behaviors are available in Table 1.

**Table 1.** Descriptive statistics, internal consistency, and Pearson correlations for all key measures

	n	Observed Range	Mean (SD)	Internal Consistency α/ω	Past Year Sports Bet Freq	Past Year Fantasy Freq	Past Year Daily Fantasy Freq	Past Year E-sports Freq
Gender (1 = Male; 0 = Other)	4363	–	–	–	.17**	.18**	.15**	.09**
Religiousness	4363	−1.52—1.44	0.51 (0.50)	.83/.84	.06**	.09**	.10**	.14**
Age	4363	19—96	0.00 (0.87)	–	−.14**	−.18**	−.19**	−.20**
NIDA ASSIST: Alcohol Score	3267	−0.56—3.38	49.56 (16.15)	.86/.86	.27**	.28**	.36**	.39**
NIDA ASSIST: Tobacco/Nicotine Score	1571	−0.96—1.94	0.00 (0.80)	.70/.71	.14**	.15**	.20**	.22**
NIDA ASSIST: Prescription Drug Misuse Score	781	−0.87—2.00	0.00 (0.68)	.85/.86	.26**	.26**	.32**	.33**
NIDA ASSIST: Marijuana Score	1564	−0.69—2.48	0.00 (0.80)	.84/.85	.27**	.25**	.36**	.36**
NIDA ASSIST: Pornography Score	2519	−0.61—2.96	0.00 (0.80)	.85/.85	.24**	.28**	.34**	.41**
NIDA ASSIST: Video Game Score	2813	−0.61—2.76	0.00 (0.79)	.86/.87	.25**	.30**	.36**	.41**
Alcohol Frequency	4363	0—5	1.81 (1.42)	–	.21**	.16**	.16**	.14**
Tobacco/Nicotine Frequency	4363	0—5	1.23 (1.86)	–	.16**	.14**	.16**	.19**
Prescription Drug Misuse Frequency	4363	0—5	0.43 (1.08)	–	.25**	.24**	.28**	.32**
Marijuana Frequency	4363	0—5	0.95 (1.54)	–	.17**	.15**	.17**	.17**
Illicit Drug Frequency	4363	0—5	0.35 (1.02)	–	.21**	.21**	.26**	.29**
Pornography Use Frequency	4363	0—5	1.42 (1.51)	–	.21**	.20**	.20**	.18**
Video Game Frequency	4363	0—5	1.95 (1.80)	–	.18**	.17**	.19**	.20**
Past Year Traditional Freq	4363	0—5	0.67 (1.36)	–		.44**	.40**	.39**
Past Year Fantasy Freq	4363	0—5	0.65 (1.41)	–			.60**	.41**
Past Year Daily Fantasy Freq	4363	0—5	0.50 (1.27)	–				.44**
Past Year e-sports Freq	4363	0—5	0.41 (1.14)	–				

Notes: *n* = number of respondents completing measure; SD = Standard Deviation; α = Cronbach’s Alpha; ω = McDonald’s Omega.

All correlations significant at the *p* < .001 level.

### 5.2. Measures

Basic demographic covariates were provided by YouGov based on panel data. We included gender, age, and overall religiousness as covariates in key analyses. Gender was coded as 1=male, 0=not-male, as small sample sizes of non-binary individuals made separating all genders unfeasible. Descriptive statistics (means, standard deviations, and observed ranges) and measures of internal consistency (Cronbach’s Alpha and McDonald’s Omega) for all measures are available in Table 1.

### 5.3. Religiousness

Religiousness was measured via the average of standardized responses to three items taken from Pew Research Center (Research Center, 2015). These items measure both the importance of religion in daily life and the presence of religious behaviors in daily life (i.e., “How important is religion in your life—very important, somewhat important, not too important, or not at all important?”; “People practice their religion in different ways. Outside of attending religious services, do you pray several times a day, once a day, a few times a week, once a week, a few times a month, seldom, or never?”; and “Aside from weddings and funerals, how often do you attend religious services … more than once a week, once a week, once or twice a month, a few times a year, seldom, or never?”). In their raw form, lower response values correspond to greater religiousness. Accordingly, upon taking the average of standardized responses to the above three questions, the average was transformed by multiplying by −1 so that higher scores corresponded to higher religiousness.

### 5.4. Sports wagering

We assessed four forms of sports wagering in the present study, corresponding to the four forms of sports wagering discussed above. Specifically, we asked participants a series of face valid questions about engagement in sports wagering. We first asked participants if they had engaged in traditional sports-wagering (i.e., bet on outcomes or specific aspects of a game), bet on e-sports (i.e., bet on competitive video game play), engaged in paid traditionally fantasy sport leagues, or engaged in paid daily fantasy sport leagues over the past 12 months. Among those who reported past-year engagement, we then asked them to rate the frequency of their engagement on a scale of 1 (*once or twice*) to 6 (*more than once per day*). This resulted in a full scale of 0 (including “no use” in the past 12 months) to 6 (indicating greater than daily use).

### 5.5. Addictive behaviors

To assess a full range of addictive behaviors, we included a modified version of the Alcohol, Smoking and Substance Involvement Screening Test (ASSIST; WHO ASSIST Working Group, 2002; Grubbs et al., 2022; National Institute on Drug Abuse, 2009). Beginning with the NIDA Quick Screen, we assessed past year frequency of alcohol use, nicotine products, prescription drugs for non-medical reasons, and illegal/illicit drugs. Additionally, given the evolution of THC-related regulations over recent years, we added a choice for cannabis/THC-related products to our focal substances, as it is no longer accurately subsumed under “illicit drugs”. In keeping with the goals of this study, we also included pornography use and gaming as response options on the NIDA-ASSIST, in addition to the substances above.

The original NIDA Quick Screen measured frequency of addictive behavior engagement in a range of *Never* to *Daily or Almost Daily*. Given that we included behaviors that likely occur multiple times per day for many users (i.e., nicotine use), we added an additional response option of *more than once per day*. Only participants who endorsed at least some past-year use on these frequency items completed further measures of problematic use.

To assess problematic or potentially addictive use of these substances, we took the average of standardized responses to problematic use questions present in the NIDA-ASSIST. Participants responded to three frequency-based questions that assess problematic use (“In the past three months, how often have you had a strong desire or urge to use or engage in the following?”, “In the past three months, how often has your use of or engagement in the following led to health, social, legal, or financial problems for you?”, and “During the past 3 months, how often have you failed to do what was normally expected of you because of your use of or engagement in the following?”) on a scale of 0 (*never*) to 5 (*more than once per day*) for each of the above mentioned substances and behaviors. Participants also responded to two questions with only three response options: “Has a friend or relative or anyone else ever expressed concern about your use of or engagement in the following?” and “Have you ever tried and failed to control, cut down or stop using or engaging in the following?” for each substance on a 3-point scale (0 = *No, Never*; 3 = *Yes, but not in the past 3 months*; and 6 = *Yes, In the past 3 months*). Given the varying response scales, responses to each of the above five items were standardized (i.e., converted to z-scores), and an average was taken for each substance or behavior of interest (i.e., alcohol, tobacco/nicotine, prescription drugs, illicit drugs, cannabis, pornography, and gambling), resulting in scale scores with a mean of 0.00 (as they were the average of z-scores) and a possible range of roughly −3.5 to +3.5.

### 5.6. Analytic plan

Pearson correlations were calculated between key measures to demonstrate bivariate associations. Given the large nature of the sample and the corresponding high degree of statistical power inherent simple correlational analyses, we elected only to highlight those associations for which a medium effect (i.e., *r* > .20; Funder & Ozer, 2019) was observed. Hierarchical regressions using a model-building approach were then conducted to determine the relationship between sports wagering and the frequency of addictive behaviors and self-reported symptoms of addiction to such addictive behaviors. For both sets of analyses, the same analytic approach was used. In the first step of the hierarchical regression, the target behavior (for both frequency and self-reported symptoms of addiction) was regressed on demographic covariates (age, gender, religiousness). The only variation between analyses was the inclusion of target behavior frequency in step 1 of the hierarchical regressions predicting self-reported symptoms of addiction to various substances. In the second step, the four sports-wagering variables were added as predictors to show their unique effect in accounting for variance in outcome variables. Specifically, the target behaviors or self-reported symptoms of addiction were regressed on the four sports-wagering frequency variables, in addition to the above-mentioned demographic covariates.

## 6. Results

Item descriptives, measures of internal consistency, and Pearson correlations for sports-wagering behaviors are reported in Table 1. Across gambling behaviors, frequency was positively correlated with almost all other key measures, with effects ranging from very small (*r*=.06, *p* < .001, between Traditional Sports Betting and Religiousness) to very large (*r*=.41, *p* < .001; between past year e-sports betting and NIDA-ASSIST Pornography scale scores) by benchmarks for correlation effect sizes in psychological research (Funder & Ozer, 2019). Focusing on those associations that were consistently of medium effect size or greater (*r* > .20; Funder & Ozer, 2019), we observed that all forms of sports wagering were positively associated with NIDA ASSIST scale scores related reflecting addictive or problematic use of alcohol, prescription drugs, and marijuana, as well as pornography viewing and video game play. Similarly, we observed positive associations in the medium range or greater between frequency of all forms of sports wagering and frequency of US prescription drug misuse, illicit drug use, and pornography viewing frequency.

### 6.1. Research question 1: associations between sports betting frequency and potentially addictive behavior frequency

With regard to behavioral frequency, hierarchical regressions revealed a consistent pattern of findings (See Table 2). In the first step of the regressions, being of younger age was universally related to higher frequency of key behaviors, and being male was generally associated with higher frequency of such behaviors. Religiousness showed differential effects, being associated with greater frequency of tobacco, prescription drug, and illicit drug use, reduced frequency of alcohol, marijuana, and pornography use, and no association with video game play. The effects of these demographic predictors were variable in size, accounting for 1% (tobacco frequency) to 7% (illicit drug frequency) of variance for target substances, and higher levels of variance (9% for video games; 27% for pornography) for behaviors.

**Table 2.** Hierarchical regression using sports-wagering frequency to predict frequency of addictive behaviors for various behaviors/substances

	Alcohol	Tobacco	THC	RX	Illicit	Pornography	Gaming
	4362	4362	4362	4362	4362	4362	4362
	*β*	*β*	*β*	*β*	*β*	*β*	*β*
Step 1							
Male Gender	.115**	.048**	.018	.039**	.027	.422**	.089**
Religiousness	−.049**	.033*	−.057**	.130**	.080**	−.104**	.028
Age	−.077**	−.099**	−.173**	−.215**	−.261**	−.274**	−.289**
*R*^*2*^	.023	.012	.035	.059	.071	.270	.090
F^†^ for *R*^*2*^	33.52	18.26	53.10	91.23	110.99	538.72	143.48
Step 2							
Male Gender	.070**	.012	−.021	−.020	−.022	.393**	.056**
Religiousness	−.078**	−.002	−.088**	.074**	.032*	−.127**	.000
Age	−.028	−.048**	−.123**	−.131**	−.188**	−.238**	−.245**
Past Year Traditional	.143**	.072**	.088**	.104**	.067**	.057**	.072**
Past Year Fantasy	.040*	.006	.030	.015	.012	.024	.026
Past Year Daily Fantasy	.049*	.066*	.063**	.123**	.120**	.047**	.050**
Past Year E-sports	.048**	.121**	.084**	.186**	.162**	.063**	.081**
*R*^*2*^	.066	.051	.072	.159	.143	.290	.117
Δ*R*^*2*^	.044	.038	.037	.100	.072	.019	.027
F^†^ for Δ*R*^*2*^	51.15	44.05	42.85	128.85	91.83	29.17	33.72

Notes: *p < .05, **p < .01.

^†^All reported F-values significant at *p* < .001. THC=Tetrahydrocannabinol, RX=Prescription medications.

In the next step of the regression (See Table 2), traditional sports wagering frequency, daily fantasy sports play frequency, and e-sports wagering frequency were each uniquely related to behavioral frequency for each target behavior or substance. Traditional fantasy sports play was largely unrelated to behavioral frequency. Variance accounted for the introduction of sports wagering variables ranged from approximately 4% (for Alcohol, Tobacco, and Marijuana) to 10% (for illicit drug use) for substances, and lower levels for target behaviors (approximately 2% for both pornography and video games).

### 6.2. Research question 2: associations between sports betting frequency and self-reported symptoms of addiction

With regard to symptoms of addiction (See Table 3), results were generally consistent across all target behaviors and substances. In the first step of the hierarchical regression, among individuals with past year use of or engagement with key substances and behaviors, greater religiousness, younger age, and greater frequency of use were each associated with greater reported symptoms of addiction. Effect sizes varied, ranging from roughly 17% of variance in self-reported symptoms of addiction (for tobacco/nicotine) accounted for by the first step of the regression to 26% (for illicit drug use).

**Table 3.** Hierarchical regression using sports-wagering frequency to predict self-reported symptoms of addiction to various behaviors/substances

	Alcohol	Tobacco	THC	RX	Illicit	Pornography	Gaming
	3266	1570	1563	780	612	2518	2812
	*β*	*β*	*β*	*β*	*β*	*β*	*β*
Step 1							
Male Gender	.023	−.075**	.054*	.038	.008	−.020	.031
**Religiousness	.158**	.167**	.271**	.173**	.277**	.299**	.228**
Age	−.299**	−.231**	−.335**	−.310**	−.194**	−.274**	−.325**
Target Behavior Frequency	.382**	.364**	.362**	.400**	.448**	.359**	.250**
*R*^*2*^	.260	.167	.336	.341	.416	.322	.219
F^†^ for *R*^*2*^	286.11	79.85	196.97	100.55	108.43	298.58	197.30
Step 2							
Male Gender	−.015	−.108**	.011	.009	−.008	−.036	−.015
Religiousness	.095**	.115**	.201**	.132**	.242**	.227**	.156**
Age	−.218**	−.158**	−.267**	−.260**	−.175**	−.208**	−.245**
Target Behavior Frequency	.322**	.361**	.335**	.359**	.423**	.328**	.211**
Past Year Traditional	.046*	.030	.061*	.062	.044	.007	.024
Past Year Fantasy	.002	.037	−.034	.034	−.003	.021	.044*
Past Year Daily Fantasy	.143**	.099**	.166**	.076*	.076*	.122**	.136**
Past Year E-sports	.200**	.116**	.145**	.106**	.050	.209**	.218**
*R*^*2*^	.343	.210	.398	.377	.429	.402	.323
Δ*R*^*2*^	.083	.040	.063	.035	.013	.080	.104
F^†^ for Δ*R*^*2*^	103.21	19.90	40.41	10.90	3.47	83.60	107.41

Notes: *p < .05, **p < .01.

^†^All reported F-values significant at *p* < .01. THC=Tetrahydrocannabinol, RX=Prescription medications.

In the second step of the regression (See Table 3), the introduction of past-year sports-wagering frequency produced similar results, with frequency of daily-fantasy play and frequency of e-sports wagering positively predicting reported symptoms of addiction for all key substances and behaviors, except for illicit drug use, for which only daily fantasy play emerged. Additionally, across almost all key substances and behaviors, regular fantasy sports play did not emerge as a significant predictor, except for a weak relationship with self-reported symptoms of addiction to gaming. Variance accounted for varied substantially among symptoms of substance addictions, accounting for roughly 1% of the variance in self-reported symptoms of addiction to illicit drugs to roughly 8% for self-reported symptoms of addiction to alcohol. For symptoms of behavioral addictions, effect sizes were generally larger, with the second step of the regression accounting for roughly 8% and 10% of the variance in self-reported symptoms of addiction to pornography and gaming, respectively.

## 7. Discussion

At the outset of this work, we evaluated links between four forms of sports wagering behaviors and other addictive behavior patterns in a U.S. census-matched sample. Though we framed the present work as largely descriptive in nature, we expected to find positive associations between sports wagering and various forms of substance use, potentially addictive behaviors, and self-reported symptoms of addiction. In general, we found that traditional sports wagering, daily fantasy sports play, and e-sports wagering were consistently associated with greater frequency of substance use across substances, as well as greater video game play and greater pornography consumption. We also found that, among participants using or engaging with key substances or behaviors, both daily fantasy sports play and e-sports wagering were uniquely associated with greater symptoms of addiction. Collectively, the findings of the present work extend past research showing that sports wagering is robustly associated with problematic or addictive behavior patterns beyond gambling problems alone (Etuk et al., 2022). That is, consistent with prior literature clearly demonstrating that gambling is a risk factor for substance use and substance use disorders (Lorains et al., 2011; Petry et al., 2018), sports wagering also appears to follow a similar path.

### 7.1. Implications

Results from the present work enhance current understandings of gambling generally and sports wagering specifically by expanding what is known about specific forms of sports wagering. That is, our findings generally demonstrate that not all forms of sports wagering are equal with regard to their links with problematic or concerning outcomes. For example, daily fantasy sports play, e-sports wagering, and traditional sports betting are clearly more concerning behaviors than general fantasy sports play. Consistent with findings regarding problem gambling itself (J. B. Grubbs & S. W. Kraus, 2022), general fantasy sports play is unlikely to be associated with other risky behavior patterns in the absence of other forms of sports wagering. Although not assessed in the current study, an important next step in this body of research is to understand whether or not active substance use (e.g., alcohol, marijuana) which occurs during sports betting behaviors influence risk taking among participants, as the links between gambling and substance use generally appear to be reciprocal and escalating (Petry et al., 2018).

Ultimately, the findings of the present work point to both the clinical and the research relevance of sports wagering behaviors. Clinically, it is clear that a large proportion of the U.S. population has recently gained access to a form of recreation with known links to a range of addictive and problematic behaviors. As access to sports wagering continues to increase across the U.S., it is quite likely that more Americans will engage with sports wagering behaviors in the coming years, particularly as sports betting ads become more increasingly commonplace across the U.S. In clinical settings, it is quite likely that sports wagering should be considered alongside a range of other potentially problematic or risk-taking behaviors such as substance use and gambling more generally. Although not all who wager on sports will do so problematically or engage in a variety of other potentially addictive behaviors, there is evidence suggesting that they will be more likely to, underscoring the importance of screening for potential gambling-related problems across a variety of healthcare settings such as primary care (Kraus et al., 2020), which could bolster early detection efforts for medical providers. Screening for gambling disorder is easily accomplished (Dowling et al., 2018) using well-validated scales such as the two-item Lie-Bet (Johnson et al., 1997) or the three-item Brief Biosocial Gambling Screen (Gebauer et al., 2010). Given the increased access to sports wagering prevalent across the U.S. and the associations documented in the present work, the regular implementation of such screeners into standard assessment in healthcare and mental-health settings seems prudent.

With regard to research, the implications of the present work are somewhat self-evident. As we have repeatedly noted throughout this work, a substantive body of research on sports wagering exists outside the U.S., with important findings consistently emerging (Etuk et al., 2022). Though such findings are of relevance in the U.S., the regulatory climate of the U.S. means that the varieties of sports betting available and the specific means of accessing sports betting are not necessarily directly comparable with other nations. Given a relatively limited body of research on sports betting in the U.S., these issues strongly suggest a need to better understand sports betting in the U.S. generally. When these general observations are taken alongside the findings of the present work linking sports betting to various potentially addictive behaviors, there is also a specific need for sports betting research in the context of a variety of health behaviors. We cannot authoritatively speculate about whether such increases in sports wagering will directly translate into increases in addictive behaviors generally, but findings of the current study highlight the need to proactively assess for such increases across the greater American population over time.

Finally, with regard to prevention efforts, the present work highlights the need for specific public health and education initiatives that aim to prevent gambling-related harm for at-risk groups. Given that sports betting individuals are more likely to be younger men, who are already prone to a range of impulsive and addictive behaviors more generally, the links between sports betting and addictive behaviors show a growing need to comprehensively address the potential harms of gambling behaviors generally, as well as sports betting behaviors specifically in adolescent and emerging adult populations. As one example, such outreach might take the form of public education initiatives in secondary school and university settings where educational materials could be developed to promote responsible approaches to gambling and help adolescents and emerging adults to identify early warning signs of problematic sports betting behaviors within themselves or their social networks.

### 7.2. Limitations

Despite the above implications, we note that the present work was based on self-reported responses to an internet-administered survey. Though YouGov is a leading polling firm with internationally acclaimed quality and representativeness, the nature of many psychiatric conditions, including symptoms and personalized experiences, reported by individuals who participated in the study may be skewed, As such, there is a clear need for more comprehensive work on these topics using more in-depth techniques such as structured clinical interviews, clinical diagnosis, and epidemiological methods. Similarly, the above listed findings are all cross-sectional in nature. Though we can certainly conclude that individuals who engage in sports wagering are more likely to report other forms of addictive behaviors, we cannot draw any temporal or causal inferences, showing a clear need for longitudinal work in the future.

## 8. Conclusions

Over recent years, access to sports wagering has increased substantially, with many Americans now living in states that allow at least some form of wagering on sports activities. Though it is likely that most individuals who wager on sports will do so without consequence or harm, there is increasing evidence that sports wagering is associated with a range of risky behaviors, including greater reported frequency of use of various substances, video game play, and pornography use. Additionally, Americans who wager on e-sports, take part in daily fantasy leagues, or gamble via traditional sports wagering seem to be at elevated risk of reporting symptoms of addiction to or dysregulation in their use of substances and potentially addictive behavior patterns. Though the present work does not speak to causality or temporality, current findings suggest that sports wagering behaviors should be an area of research interest and potential clinical concern.

## CRediT statement

JBG and SWK were responsible for the conception and design of this project, and data collection. JBG developed the analytic plan; SWK provided feedback on the analytic plan. JBG conducted all formal data analysis. JBG and SWK wrote, edited, and revised the initial draft of this manuscript.

## Data policy

Data are available at public repository (https://osf.io/283ys/).
